# mGem: AAV, from almost a virus to an awesome vector—or is it?

**DOI:** 10.1128/mbio.02957-25

**Published:** 2026-01-05

**Authors:** Arun Srivastava

**Affiliations:** 1 Division of Cellular and Molecular Therapy, Department of Pediatrics, Powell Gene Therapy Center, University of Florida College of Medicine 12233, Gainesville, Florida, USA; 2 Department of Molecular Genetics & Microbiology, University of Florida College of Medicine, Gainesville, Florida, USA; Albert Einstein College of Medicine, Bronx, New York, USA

**Keywords:** adeno-associated virus vectors, gene transfer, transgene expression, gene therapy

## Abstract

Adeno-associated virus (AAV) vectors have taken center stage for gene therapy and have shown clinical efficacy in 15 human diseases to date. The Food and Drug Administration has approved seven AAV “drugs” for one-time treatment respectively for Leber’s congenital amaurosis, spinal muscular atrophy, hemophilia B, Duchenne muscular dystrophy, hemophilia A, and aromatic L-amino acid decarboxylase deficiency. Despite these remarkable developments, it has become increasingly clear that the first generation of AAV vectors is less than optimal since in most, if not all, cases, exceedingly high doses are needed to achieve clinical efficacy, and as a consequence, in some patients, serious adverse events have been observed, and to date, at least 21 patients have died. Thus, there is a need to reassess the limitations of the first generation of AAV vectors as well as an urgent need to develop the next generation of AAV vectors that are safe and effective.

## PERSPECTIVE

Adeno-associated virus (AAV), discovered in 1965 (1), was considered a “biological oddity” and was dubbed as “almost a virus” (2), because it fails to undergo a productive replication in the absence of co-infection with a helper virus, such as adenovirus (3), herpesvirus (4), vaccinia virus (5), or human papillomavirus (6). Infection with the wild-type (WT) AAV infection is also not associated with any known disease in humans. However, following the availability of the complete nucleotide sequence of the WT AAV2 genome (7), molecular cloning (8, 9), and the demonstration of its remarkable ability to integrate site-specifically into human chromosome 19q13.3 (10, 11), sparked a significant interest in AAV, subsequently leading to the development of the first generation of recombinant AAV2 vectors (12, 13), followed by further refinements (14, 15).

Ever since then, interest in AAV vectors has continued to grow exponentially (16–19). The first generation of AAV vectors has been used in at least 700 programs, and there are over 200 currently active Phase I/II/III clinical trials for gene therapy of a wide variety of human diseases. Thus far, seven AAV “drugs”—Luxturna for Leber congenital amaurosis (20–22); Zolgensma for spinal muscular atrophy (23); Hemgenix for hemophilia B (24–27); Elevidys for Duchenne muscular dystrophy (28); Roctavian for hemophilia A (29–32); Beqvez for hemophilia B (33) (Beqvez has now been discontinued); and Kebilidi for aromatic L-amino acid decarboxylase deficiency (34, 35)—have been approved by the US Food and Drug Administration. A large number of AAV serotype vectors have also been generated (36, 37), which have shown clinical efficacy in several additional human diseases, such as lipoprotein lipase deficiency (38), choroideremia (39), Tay-Sachs disease (40), Crigler-Najjar syndrome (41), giant axonal neuropathy (42), hereditary spastic paraplegia type 50 (43), Leber’s congenital amaurosis type 1 (44), and infantile-onset Pompe’s disease (45).

Despite these remarkable advancements, I have posited that the use of the first generation of AAV vectors in humans is unlikely to be optimal for the simple reason that AAV evolved as a virus, and not as a vector for the purpose of delivering therapeutic genes (46–48). This has been borne out by the fact that these vectors have either shown lack of efficacy, only limited efficacy in some cases, and in nearly all cases, serious adverse events, especially at high dose systemic delivery, leading to 21 deaths to date (49). These details are provided in Table 1.

**TABLE 1:** Reported cases of lack of efficacy, serious adverse events, and deaths, associated with the use of first generation of AAV vectors in various human diseases^*c*^

Sponsor	Disease	Serotype	Vector dose	Observations/adverse events	Outcome
Amicus Therapeutics	BD	AAV9	5 × 10^13^ vgs/kg	Lack of efficacy (50)	Terminated
Baxalta	HB	AAV8	3 × 10^12^ vgs/kg	Efficacy in only one patient (51)	Terminated
Solid Biosciences	DMD	AAV9	3 × 10^14^ vgs/kg	Thrombocytopenia, renal failure, cardio-pulmonary insufficiency (52)	
Audentes Therapeutics	XLMTM	AAV8	3 × 10^14^ vgs/kg 1 × 10^14^ vgs/kg	Acute liver toxicity (53)	3 deaths 1 death
Lysogene	MPS-IIIA	AAVrh10	7 × 10^12^ vgs/kg		1 death^*a*^ (54)
Pfizer	DMD	AAV9	2 × 10^14^ vgs/kg	Acute kidney injury, atypical hemolytic uremic syndrome-like complement activation, thrombocytopenia (55)	2 deaths
Novartis	SMA	AAV9	1.1 × 10^14^ vgs/kg	Acute liver toxicity (56, 57)	8 deaths
Cure Rare Disease	DMD	AAV9	1 × 10^14^ vgs/kg	Acute respiratory distress syndrome (58)	1 death
Neurogene	RS	AAV9	3 × 10^15^ vgs [total]	Hemophagocytic lymphohistiocytosis (59)	1 death
Sarepta Therapeutics	DMD LGMD	AAVrh74	2 × 10^14^ vgs/kg	Acute liver toxicity (60, 61)	2 deaths 1 death
Rocket Pharma	DD	AAV9	6.7 × 10^13^ vgs/kg	Capillary leak syndrome (62)	1 death^*b*^

^*a*^ The exact cause of death unknown (5 months post-gene therapy).

^*b*^ The exact cause of death unclear (either due to the use of the immune-suppressant drug Empaveli, or AAV9, or both).

^*c*^ BD, Batten disease; HB, hemophilia B; DMD, Duchenne muscular dystrophy; XLMTM, X-linked myotubular myopathy; MPSIIIA, mucopolysaccharidosis type IIIA; SMA, spinal muscular atrophy; RS, Rett syndrome; LGMD, limb-girdle muscular dystrophy; DD, Danon disease.

So, the question must be asked as to why is this happening? The obvious answers are twofold. First, in every case, vectors based on AAVs derived from non-human primates have been used. These vectors, although safe and efficient in mice and in monkeys, are not so in humans since, simply put, humans are not large mice and monkeys. And second, doses ranging from 3 × 10^12^ vector genomes per kilogram of body weight (vgs/kg) (2.1 quadrillion) to as high as 3.8 × 10^14^ vgs/kg (26.6 quadrillion) have been used, which translates to a total AAV vector particle numbers per cell in the human body ranging from 7 to 866, assuming an average human body weight of 70 kg, composed of 3 × 10^13^ (30 trillion cells) (63). These are remarkably high vector doses, indeed! Thus, there is little doubt that the use of high vector doses correlates directly with the documented adverse events in patients.

The next question is why are such high vector doses needed to achieve clinical efficacy? The answer is intuitive, yet has been overlooked to some extent. AAV, as a virus, is not a particularly efficient virus. So, the first generation of AAV vectors composed of naturally occurring capsids cannot be expected to be very efficient either. For example, much the same way as the WT AAV, a large fraction of the first generation of AAV vectors has been shown to undergo phosphorylation at surface-exposed tyrosine (64–66), serine (67), and threonine (68) residues, which serves as a signal for ubiquitination at lysine (69) residues, followed by proteasome-mediated degradation (70, 71), which negatively impacts not only on the transduction efficiency, but the broken down peptides also trigger a cytotoxic T-cell response (72).

The next obvious question is what can be done to overcome the limitations of the first generation of AAV vectors? Several elegant approaches have been employed by a number of investigators to generate more efficient AAV vectors, which include the following: peptide insertions (73–76), directed evolution (77–80), DNA shuffling (81–83), rational design (66–69, 71, 84–86), ancestral reconstruction (87, 88), chimeric vectors (89, 90), dual vectors (91–94), protease activation (95, 96), chemical modifications (97, 98), and machine learning (99, 100). Whereas these strategies have led to the development of AAV serotype vectors that are remarkably efficient in transducing various tissues and organs in preclinical animal models, it has become increasingly clear that none of these animal models is a true surrogate for, or a predictor of, therapeutic efficacy of these vectors in human diseases since the tropisms of these vectors in animal models do not necessarily translate well in humans (101).

Extensive time, effort, and resources have been, and continue to be, expended in AAV capsid-engineering, but have led to diminishing returns, for the simple reason that extensive modifications in the capsid likely lead to creation of hitherto unknown completely novel antigenic epitopes, both T and B cell-specific, which may be tolerated well in animals but lead to increased immunogenicity in humans. Serious adverse events in several patients, as well as the death of one patient, have occurred with the use of AAV vectors derived from directed evolution/high-throughput selection, which are detailed in Table 2.

**TABLE 2:** Adverse events and death reported with AAV vectors derived from directed evolution/high throughput selection in various human diseases^*b*^

Sponsor	Disease	Variant	Vector dose	Symptoms/outcome
Adverum	DME	AAV.7m8	6 × 10^11^ vgs/eye	Loss of significant vision; 6 patients (102)
Spark Therapeutics	HA	AAV-LK03	2 × 10^12^ vgs/kg	Capsid immune response; 2 patients (103)
4D Molecular Therapeutics	FD	4D-310	3 × 10^13^ vgs/kg	Transient atypical hemolytic uremic syndrome; 3 patients (104)
LogicBio Therapeutics	MMA	AAV-LK03	5 × 10^13^ vgs/kg	Thrombotic microangiopathy; 2 patients (105)
Capsida Biotherapeutics	STXBP1-DEE	CAP-002	3.8 × 10^13^ vgs/kg^*a*^	Acute respiratory distress syndrome, cardiac arrest, and death (106)

^*a*^ Not disclosed, but based on the lowest dose used in non-human primates.

^*b*^ DME, diabetic macular edema; HA, hemophilia A; FD, Fabry disease; MMA, methylmalonic acidemia; STXBP1-DEE, syntaxin-binding protein 1 developmental and epileptic encephalopathy.

A second limitation of most of the first generation of AAV vectors currently being used in clinical trials should also have been obvious and, yet again, did not receive adequate attention. As stated above, AAV, as a virus, does not express its own genes efficiently since the viral genome is a single-stranded DNA, which is transcriptionally inactive (107–111). In other words, viral second-strand DNA synthesis is needed before gene expression can occur since there is no host cell RNA polymerase that can transcribe a single-stranded DNA genome. Since AAV inverted terminal repeats (ITRs) are known to play a significant role in regulating viral second-strand DNA synthesis (112), most of the single-stranded AAV vectors currently in use are also sub-optimal in expressing therapeutic genes since vector second-strand DNA synthesis is needed before gene expression can occur. Thus, the expectation that single-stranded AAV vectors express therapeutic genes to high levels is also unrealistic.

So, what needs to be done to overcome the first limitation of the first generation of AAV vectors? The obvious answer is to modify the naturally occurring capsid, but to keep the extent of these modifications to a minimum such that the vector is different from the virus, and yet does not trigger the immune response—the Goldilocks solution! (112). Indeed, one such minimally capsid-modified AAV2 vector has shown clinical efficacy in a Phase I/II clinical trial for gene therapy of Leber hereditary optic neuropathy (LHON), with no serious adverse event (113). However, beyond LHON, targeting the somewhat immune-privileged nature of the eye, additional clinical trials with such capsid-modified AAV serotype vectors in other disease targets will be needed to support the use of these vectors for gene therapy in humans (101).

Similarly, the AAV-ITRs must also be modified in order to achieve higher levels of transgene expression (114, 115). Combining capsid and ITR modifications has been reported to lead to the development of optimized AAV serotype vectors that are more efficient at significantly lower doses (116, 117). The lower vector dose per patient may, in some cases, also obviate the need for prophylactic immune suppression that is frequently used in most, if not all, current AAV gene therapy trials, and which has its own potential complications (118). However, it is likely that prophylactic immune suppression may still be necessary in some cases, even with low-dose AAV delivery. Lower vector dose per patient will also allow more patients to be treated than high vector doses that are currently being used. Thus, instead of treating one patient, 10, or maybe 100 patients could be treated, which would not only bring down the cost per patient but also reduce the vector production costs.

An additional aspect that also deserves serious discussion, pertains to the tropism of AAV vectors. For instance, preclinical evaluation of safety and efficacy of AAV vectors in animal models has not always translated well in humans (101). As detailed in Table 1, vectors based on AAVs derived from non-human primates were observed to be safe and effective in mice and in monkeys but led to serious adverse events and in deaths of several patients. Thus, it is of paramount importance to develop the next generation of human-tropic AAV vectors that are capable of transducing primary human cells and organs. Work from my laboratory has identified two such AAV vectors, AAV3 and AAV6, that selectively and efficiently transduce primary human hepatocytes (119, 120) and primary human hematopoietic stem cells (121, 122), respectively. Capsid-modified AAV3 vectors have also been shown to be significantly more efficient in primary human hepatocytes in “humanized” mice (123–125) as have capsid-modified AAV6 vectors in primary human hematopoietic stem cells (126, 127).

Even the first generation of AAV6 vectors has been shown to be efficient in transducing primary human hematopoietic stem cells (128–131), primary human T cells (132), primary human mesenchymal stromal cells (133), primary human natural killer cells (134), primary human induced pluripotent stem cells (135), and primary human alveolar stem cells (136).

Thus, the answer to the question posed by the title of this article is that AAV vectors can, and will, prove to be awesome, provided:

- The use of the first generation of AAV serotype vectors composed of naturally occurring capsids is avoided.
- The extent of the capsid modification is kept to a minimum to make AAV vectors different from the WT AAV, to minimize triggering the host immune response.
- The use of the first generation of AAV serotype vectors containing the WT-ITRs is avoided.
- The total AAV vector doses used do not exceed 1 × 10^13^ vgs/kg.
- Capsid + ITR-modified AAV serotype vectors are used in future clinical trials.

Further optimizations of capsid + ITR-modified AAV serotype vectors that are human-tropic, safe and efficient at lower doses, and cost-effective, will continue to revolutionize gene therapy of a wide range of human diseases, hopefully, in the not too distant future.
